# Diet reveals links between morphology and foraging in a cryptic temperate reef fish

**DOI:** 10.1002/ece3.3604

**Published:** 2017-11-15

**Authors:** Natalia S. Winkler, Maite Paz‐Goicoechea, Robert W. Lamb, Alejandro Pérez‐Matus

**Affiliations:** ^1^ Subtidal Ecology Laboratory and Marine Conservation Center Estación Costera de Investigaciones Marinas Facultad de Ciencias Biologicas Pontificia Universidad Católica de Chile Santiago Chile; ^2^ Department of Ecology and Evolutionary Biology Brown University Providence RI USA

**Keywords:** diet, fish, foraging, kelp forest, microhabitat, morphological structures, phenotypic plasticity, temperate reefs

## Abstract

Predators select prey so as to maximize energy and minimize manipulation time. In order to reduce prey detection and handling time, individuals must actively select their foraging space (microhabitat) and populations exhibit morphologies that are best suited for capturing locally available prey. We explored how variation in diet correlates with habitat type, and how these factors influence key morphological structures (mouth gape, eye diameter, fin length, fin area, and pectoral fin ratio) in a common microcarnivorous cryptic reef fish species, the triplefin *Helcogrammoides cunninghami*. In a mensurative experiment carried out at six kelp‐dominated sites, we observed considerable differences in diet along 400 km of the Chilean coast coincident with variation in habitat availability and prey distributions. Triplefins preferred a single prey type (bivalves or barnacles) at northern sites, coincident with a low diversity of foraging habitats. In contrast, southern sites presented varied and heterogeneous habitats, where triplefin diets were more diverse and included amphipods, decapods, and cumaceans. Allometry‐corrected results indicated that some morphological structures were consistently correlated with different prey items. Specifically, large mouth gape was associated with the capture of highly mobile prey such as decapods, while small mouth gape was more associated with cumaceans and copepods. In contrast, triplefins that capture sessile prey such as hydroids tend to have larger eyes. Therefore, morphological structures co‐vary with habitat selection and prey usage in this species. Our study shows how an abundant generalist reef fish exhibits variable feeding morphologies in response to the distribution of potential habitats and prey throughout its range.

## 1 INTRODUCTION

Feeding abilities are tightly linked to prey characteristics, and dictate the effective use of different prey types, offering a causal link between functional morphology and prey consumption and utilization patterns. In fishes, comparative morphology has revealed how diversification of these evolutionary and ecological attributes runs in parallel (Huckins, 1997; Liem & Sanderson, 1986). Morphological adaptations affect handling time and success rate for different prey types, and the distribution of these prey throughout a habitat predict the distribution of predators (Ferry‐Graham, Bolnick, & Wainwright, 2002). However, prey availability is just one of myriad factors influencing the distribution of species, which also include abiotic gradients, spawning sites, and refuges from predators (Flaxman & Lou, 2009; Fortin et al., 2005; Hammond, Luttbeg, & Sih, 2007; Iwasa, 1982). As these factors vary throughout a species’ geographic range, it is unclear how the respective distributions of suitable habitat and preferred prey types shape the way individuals move, forage, and utilize morphological elements to catch and consume prey. Previous research has revealed that when individuals face different microhabitats arranged in a heterogeneous landscape, this can yield divergent phenotypes according to local conditions and food sources (Hegrenes, 2001; Wainwright, Osenberg, & Mittelbach, 1991; Wimberger, 1992). In particular, highly diverse environments harbor a greater diversity of available prey types, potentially enhancing intraspecific foraging polymorphisms.

In addition to accurate habitat selection and prey choice, the morphological structures used in the feeding process are key factors determining the diet of a species (Ferry‐Graham, Wainwright, & Bellwood, 2001; Horn & Ferry‐Graham, 2006; Wainwright, Bellwood, & Westneat, 2002). Morphological adaptations comprise multiple structures that influence the chances of successful foraging in any given habitat (Binning & Roche, 2015). These adaptations can involve structures used for the detection, capture, or ingestion of prey. In marine fishes, for instance, mouth gape and body size directly limit the consumption of different food types (Scharf, Juanes, & Rountree, 2000; Wainwright & Richard, 1995). Ocular morphology is fundamental to prey searching (i.e., visual detection of prey) (Job & Bellwood, 2000), while differences in fin morphology affect prey acquisition (i.e., swimming speed) (Fulton & Bellwood, 2005). Thus morphological variation may suggest differential prey use and provide evidence for diverging intraspecific habitat associations (Ramírez, Pérez‐Matus, Eddy, & Landaeta, 2013).

Marine temperate environments are known for high structural complexity with a wide range of microhabitats available (Connell & Irving, 2008; Taylor, 1998), resulting in a number of possible alternative feeding strategies employed to gain a higher energetic benefit. For reef fishes in these heterogeneous environments, the number and type of potential prey differ according to habitat, making accurate selection of a foraging patch a critical determinant of evolutionary fitness (Holbrook & Schmitt, 1984). Given the distribution of available habitat and prey types, feeding behaviors and morphologies are selected for in order to reach optimal foraging by maximizing the energy consumed per unit of handling time (MacArthur & Pianka, 1966).

On temperate rocky reefs, the understory assemblage that occurs sympatrically with kelp may strongly influence the diet of fauna living in kelp beds (Angel & Ojeda, 2001; Palma & Ojeda, 2002; Pérez‐Matus, Pledger, Diaz, Ferry, & Vasquez, 2012). Crustose coralline algae (CCA), for instance, is one of the dominant understory species in kelp habitats (Connell, 2003; Melville & Connell, 2001). Crustose coralline algae may comprise more than 80% of benthic cover, reducing microhabitat complexity and understory habitat diversity while decreasing the availability of potential prey for small cryptic predators (Palma & Ojeda, 2002; Pérez‐Matus, Ferry‐Graham, Cea, & Vasquez, 2007; Pérez‐Matus et al., 2012; Taylor, 1998). On the other hand, turf or foliose understory algae are known to be important for reef‐associated fishes, as these types provide three‐dimensionally complex habitat for epifauna upon which fish forage (Holbrook, Schmitt, & Ambrose, 1990). In a recent study, (Pérez‐Matus, Sánchez, González‐But, & Lamb, 2016) described how a common cryptic reef fish discriminates among biogenic habitats and avoids dense kelp, probably due to increased predation risk in dense kelp stands. In addition, individual fish tend to prefer one microhabitat at any given site, but the preferred habitat type differs significantly between sites as a result of predation risk and food availability (Pérez‐Matus et al., 2016). The differences in habitat selection between populations of this species may be associated with polymorphisms in response to variation in resources available at each site.

Here, we tested the hypothesis that differences in the availability of foraging microhabitat influences predatory fish feeding morphology by filtering the type and number of available prey. We expected that differences in microhabitat availability in kelp beds (i.e., number and type of available microhabitats) would yield variation in available prey. This in turn should influence diet, which may produce variation in the morphological structures used for feeding by the small cryptic predatory fish *Helcogrammoides cunninghami*. Specifically we aimed to (1) describe the microhabitat availability of this temperate reef fish at different kelp‐dominated sites located in northern‐central Chile, (2) determine the diet composition of the species at each site, (3) determine morphological structures used in prey capture and assimilation, and d) relate differences in predator morphology with prey distributions.

## 2 MATERIALS AND METHODS

### 2.1 Focal species and study sites

**Figure 1:**
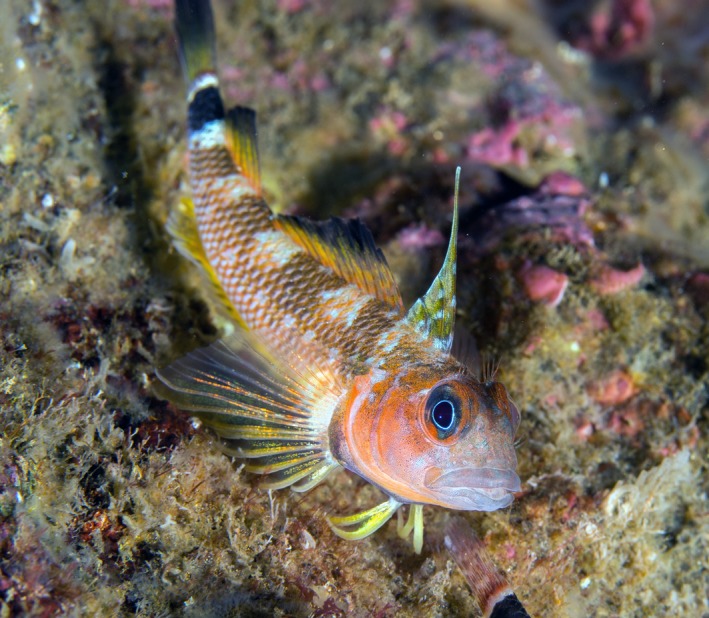
Focal species (*Helcogrammoides cunninghami*: Tripterygiidae). Photo credits: Jose Tomas Yakasovic, subelab

To test the effects of differences in microhabitat and prey availability on morphological structures used for foraging, we used the temperate reef fish family Tripterygiidae, which are small teleosts of which only two species from the *Helcogrammoides* genus are present in Chile (Williams & Springer, 2001). *Helcogrammoides chilensis* and *Helcogrammoides cunninghami* are both common and abundant species found sympatrically along the east coast of the Pacific Ocean from Peru to the south of Chile (Williams & Springer, 2001). Together they inhabit shallow waters along high‐energy exposed rocky coasts and can be found in intertidal and subtidal habitats, respectively. *H. cunninghami,* commonly known as trombolito tres aletas or Cunningham's triplefin, is a microcarnivorous and cryptic fish that is very abundant along the northern‐central Chilean coast, associated with particular subtidal reef microhabitats that vary at the local scale (Pérez‐Matus et al., 2016); Figure 1).

**Figure 2:**
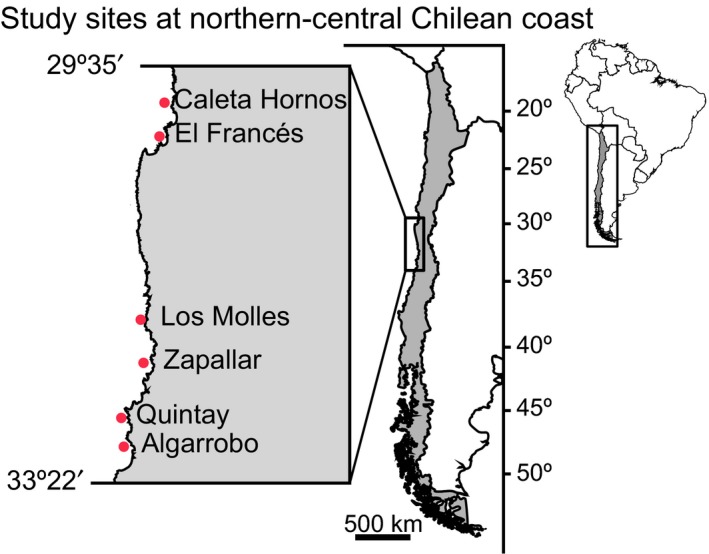
Study sites distributed along the northern‐central Chilean coast. From north to south: Caleta Hornos (CH), El Francés (EF), Los Molles (LM), Zapallar (Z), Quintay (Q), and Algarrobo (A)

Sampling of *H. cunninghami* triplefin was carried out at six different sites along the northern‐central Chilean coast ranging from 29°S to 33°S. Sites surveyed from North to South were Caleta Hornos, El Francés, Los Molles, Zapallar, Quintay, and Algarrobo (Figure 2), hereafter referred to as CH, EF, LM, Z, Q, and A, respectively. All sites are dominated by rocky substrate (medium to large boulders and cobbles), face southeast, and are relatively sheltered from wave action. All sites are dominated by the kelp *Lessonia trabeculata*, which forms a dense subtidal forest with a diverse and complex understory of corticated, foliose, filamentous, calcareous, and noncalcareous algae together with bryozoans and sponges (Pérez‐Matus et al., 2016).

In order to estimate potential differences in microhabitat availability, we quantified percent cover of the different benthic substrates available and associated sessile organisms by classifying them into four broad categories: encrusting algae, sand, sessile animals, and macroalgal understory. At each site, divers swam along two 100 m transects running perpendicular to the coast; quantifying depth (m) and benthic cover types every 10 m (n = 40). For each measurement, the diver would place a 50 × 50 cm (0.25 m^2^) quadrat with 90 equidistant intersection points on the substrate. The diver then recorded the type of cover under each intersection point for relative percent cover. Sampling was conducted between 5 and 25 m during March–April 2012.

### 2.2 Triplefin sampling

A total of 65 *Helcogrammoides cunninghami* individuals were captured at the different sampling sites between March 27 and April 10, 2012. Divers collected individuals using a 20 × 20 cm, 1 mm hand‐net along a 100 m transect, noting the associated microhabitat at time of capture. Individuals were captured in the same area as where the estimates of microhabitat availability were made. Once on shore, individuals were anesthetized with benzocaine (98% ethyl 4‐aminobenzoate), stored on ice, and transported to the Estación Costera de Investigaciones Marinas at Pontificia Universidad Católica de Chile (ECIM), where they were analyzed for morphology and gut contents. Sample processing and gut content analyses were conducted in the laboratory using dissecting and stereo microscope M125 C Leica © (Leica Microsystems) over a 35‐day period following field collections. All items were easily identified, and no signs of deterioration of prey items were detected.

### 2.3 Morphology

We quantified morphological characteristics of *H. cunninghami* that may be associated with particular prey items (Ferry‐Graham et al., 2001; Goatley & Bellwood, 2009; Horn & Ferry‐Graham, 2006). We measured total length (TL), mouth gape (MG), eye diameter (ED), pectoral fin area (PA), pectoral fin length (PL), and weight (W). Mouth gape was measured as the largest internal horizontal distance in the oral jaws that could be measured without visible distortion of the mouth. Eye diameter was measured as the maximum external width of the eyeball along the anterior–posterior axis (Goatley & Bellwood, 2009). All measurements were made using digital calipers with a precision of 0.05 mm. All individuals were weighed using a balance of 0.01 g precision. Finally, we measured fin aspect ratio (AR) by excising the fin along the proximal base of the fin rays before using fixative. Aspect ratio was calculated as the square of the leading edge of the fin length divided by the projected area of the pectoral fin. To allow comparisons with aspect ratios of other organisms for which AR is calculated using the area of both pectoral fins, we multiplied AR values by two. Thus, AR values include the area of both pectoral fins, but not the width of the body between the fins (Wainwright et al., 2002).

### 2.4 Dietary analysis

As triplefins lack a true stomach (Silberschneider & Booth, 2001), we examined the entire digestive tract. Prey items were removed from the digestive tract and placed in Petri dishes, then separated and identified to the lowest taxonomic resolution possible using a dissecting microscope. For site comparisons, we then categorized all prey items into 16 different categories of prey; hydrozoa, mollusca (other), vermetidae, bivalvia, gastropoda (other), crustacea (other), tanaideacea, isopoda, amphipoda, decapoda (other), cirripedia, cumacea, polychaeta, copepoda, ostracoda, and ophiuroidea. In order to determine if an adequate number of samples (fish individuals) had been collected to precisely describe the diet of the species, we used cumulative prey curves for each sampling site (Figure S1). We plotted the cumulative number of prey types against the cumulative number of guts analyzed (Ferry & Cailliet, 1996), which reached an asymptote for each site.

All individuals collected had food items in their guts and, previous to any measurement, we calculated the satiety index (SI) for each individual fish using a 0 to 1 scale [where 0 indicates that the alimentary tract was completely empty and 1 that it was completely full (Platell & Potter, 2001; Hynes, 1950)] by accumulating all the dietary items from each individual in a graduated petri dish. We then estimated the diet of each fish by determining the relative abundance of prey items. This method measures the total volume and number of each prey type relative to all prey consumed, expressed as a percentage (Hyslop, 1980).

A total of three measurements: volume (%V), number (%N), and frequency of occurrence (%FO) were calculated for each triplefin in order to assess the Index of Relative Importance (IRI) of each dietary category. First, we estimated the relative prey volume (%V) (Krebs, 1999) of each dietary category. The total diet volume of each sample was estimated visually in graduated petri dishes at 25 intersection points of an overlaying grid and then expressed using the point count method (Hynes, 1950; Hyslop, 1980). We also calculated the number (%N) as a proportion of the number of prey in each category divided by the total number of prey recovered from each digestive tract. All prey items were scored using a presence index (PI) according to presence in the sample of stomach contents for each individual fish on a scale of 0 to 10; where 0 indicates an absence of the prey item and 10 indicates that all of the stomach contents corresponded to that prey item (Feary, Wellenreuther, & Clements, 2009). Finally, using the satiety and presence indices, the relative prey volume was obtained as:

V%=(SI×PI)×10;

where SI represents the satiety index and PI the presence index (points method).

Third, to evaluate the relative importance of prey categories in triplefin diets, the frequency of occurrence (%FO) of each prey item was calculated as the percent occurrence of each prey item represented in the digestive tracts:

$$\%\text{FO}=\frac{S_{\mathrm{PI}}}{S_{T}}\times 100;$$

where *S*_PI_ is the number of digestive tracts in which the prey item was present and *S*_T_ is the total number of digestive tracts analyzed per site (Hyslop, 1980).

With the above information, we were able to assess the contribution of the various prey items in the diet of *H. cunninghami* by calculating the Index of Relative Importance (IRI) (Pinkas, Oliphant, & Iverson, 1971), which integrates all measurements of prey categories including the volume, the number of prey items, and frequency of occurrence:

$$\text{IRI}=\left(\%N+\%V\right)\times \%\text{FO}.$$

The IRI method is useful to infer the importance of prey categories in the diet of a species as its score depends on the volume, number, and frequency of each prey item observed among multiple individuals. Thus the IRI represents a combination of the three methods, smoothing their biases and easing our interpretation of the important prey items in the diet of this species.

### 2.5 Data analysis

We assessed differences between study sites in terms of cover of each of the four microhabitat categories and the changes in depth using univariate analyses of variance (ANOVA) and a posteriori Tukey's test. To meet ANOVA assumptions, microhabitat cover data were square‐root transformed and tested for normality and homogeneity of variances using Shapiro–Wilk's test, Q–Q plots, and Levene's test.

We measured several morphological characteristics including total length in cm (TL), standard length in cm (SL), mouth gape in mm (MG), eye diameter in mm (ED), weight in g (W), both pectoral fin areas in cm^2^ (PA), and pectoral fin length in cm (PL), which were measured to calculate pectoral fin aspect ratio (AR). We removed allometric effects by standardizing the measurements of each morphological character to the total length (TL) of the individual being measured using a power function in the following form,

$$Y_{i}^{*}=y_{i}{\left|\frac{X_{0}}{X_{i}}\right|}^{b}$$

where $Y_{i}^{*}$ is the predicted value of *Y* for the individual *i* after correcting for the relationship between *Y* and *X*;* x*_*i*_ and *y*_*i*_ are the observed values of *X* and *Y* for each individual *i*. The parameter *b* is the slope from the ordinary least squares (OLS) regression of log‐transformed *Y* and *X* variables, and *X*_0_ is the mean value of *X* (in this case, mean TL) (Lleonart, Salat, & Torres, 2000).

The relationship between the allometry‐corrected measurements of MG and TL, ED and MG, and both cranial structures (MG and ED) and PA was determined by independent correlation analyses (Pearson). In addition, we tested the effect of study site on TL, W, and allometry‐corrected measurements AR, PA, PL, MG, and ED using ANOVA.

A multivariate analysis was conducted in order to evaluate differences in diet composition among sites. Data consisted of relative volume (%*V*) of the 16 prey categories (diet composition) from 65 individuals taken from six sites. The data were square‐root transformed and analyzed according to the one‐factor (Site) experimental design using permutational multivariate analysis of variance on a Bray–Curtis dissimilarity index with 9999 permutations (PERMANOVA, Anderson, 2001). A percentage similarity analysis (SIMPER) was used to estimate the relative contributions of each prey type for the differences between sites.

Finally, a linear mixed effect model (LMM; lme4 package in R) was used to compare the effect of relative volume (%V) of each prey item on each morphological character (fixed effect), while controlling for site (random factor).

## 3 RESULTS

### 3.1 Microhabitat cover

**Figure 3:**
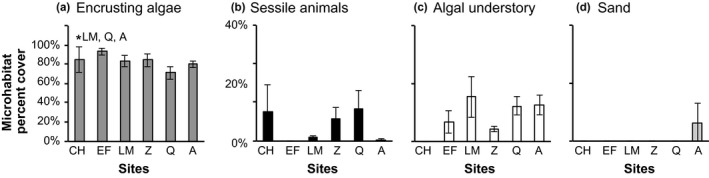
Percentage of microhabitat cover ± *SE* of (a) encrusting algae, (b) sessile animals, (c) macroalgal understory, and (d) sand at the different sampling sites: Caleta Hornos (CH), El Francés (EF), Los Molles (LM), Zapallar (Z), Quintay (Q), and Algarrobo (A). Significant differences (*p* < .05) between sampling sites are indicated with “*”

Microhabitat availability was dominated across all sites by encrusting algae, including both calcareous and noncalcareous forms (CCA: *Lithothamnium* spp. and *Hildenbrandia* spp.), which constituted between 70% and 93% of cover (Quintay and El Francés, respectively) (Figure 3). Most sites comprised three or more microhabitats including encrusting algae and both macroalgae and sessile animal understory. However, a posteriori Tukey's test following the ANOVA analyses showed that Caleta Hornos held the lowest diversity of microhabitats, as algal understory was not present (*F*_5,11_ = 6.22, *p *=* *.02) (see Figure 3 and Table S1 for details). We found that depth influenced the percent (%) cover of encrusting algae (*F*_3, 30_ = 3.0034, *p *=* *.0459) only. The posteriori Tukey's test following the ANOVA revealed that encrusting algal cover was significantly lower at depths below 20 m among sites. There was no significant interaction between site and depth for any understory microhabitats; encrusting algae (*F*_10, 15_ = 0.341, *p *=* *.954), sand (*F*_10, 15_ = 0.639, *p *=* *.76), sessile animal (*F*_10, 15_ = 0.656, *p *=* *.747), and macroalgal understory (*F*_10, 15_ = 1.061, *p *=* *.444).

### 3.2 Triplefin morphology

A total of 65 individuals from six different sites along the northern‐central coast of Chile were collected. Table 1 summarizes the average length, weight, and fin aspect ratio for fish collected at each sampling site. Length and weight of sampled individuals ranged from 2.8 to 6.2 cm and from 0.14 to 1.8 g respectively, while fin aspect ratio ranged from 0.60 to 1.29. Fish length, weight, and fin aspect ratio did not differ between sites, whereas pectoral fin length and cranial structures (mouth gape and eye diameter) differed between sites (Figure S2 and Table S2).

**Table 1:** Number of triplefin samples, mean total length (TL) (cm), mean weight (W) (gr), mean fin aspect ratio (AR), pectoral fin length (PL) (mm), pectoral fin area (PA) (cm^2^), mouth gape (MG) (mm), and eye diameter (ED) (mm) per sampling site

Site	No of samples	Total length ± *SE*	Weight ± *SE*	Fin aspect ratio ± *SE*	Pectoral fin length ± *SE*	Pectoral fin area ± *SE*	Mouth gape ± *SE*	Eye diameter ± *SE*
Caleta Hornos	8	4.06 ± 0.28	0.61 ± 0.12	1.22 ± 0.19	0.63 ± 0.04	0.1 ± 0.03	6.72 ± 0.57	2.32 ± 0.03
El Francés	9	4.16 ± 0.28	0.56 ± 0.05	1.89 ± 0.22	0.55 ± 0.04	0.14 ± 0.03	7.05 ± 0.63	2.17 ± 0.04
Los Molles	15	4.02 ± 0.25	0.57 ± 0.04	1.03 ± 0.1	0.71 ± 0.04	0.11 ± 0.01	6.14 ± 0.32	2.26 ± 0.02
Zapallar	7	4.16 ± 0.24	0.63 ± 0.18	0.88 ± 0.08	0.73 ± 0.04	0.1 ± 0.02	6.63 ± 0.65	2.27 ± 0.03
Quintay	13	4.20 ± 0.22	0.61 ± 0.12	1.30 ± 0.23	0.73 ± 0.05	0.21 ± 0.04	5.54 ± 0.28	2.29 ± 0.04
Algarrobo	13	3.95 ± 0.22	0.49 ± 0.06	1.74 ± 0.46	0.66 ± 0.03	0.13 ± 0.02	5.18 ± 0.33	2.2 ± 0.03

**Figure 4:**
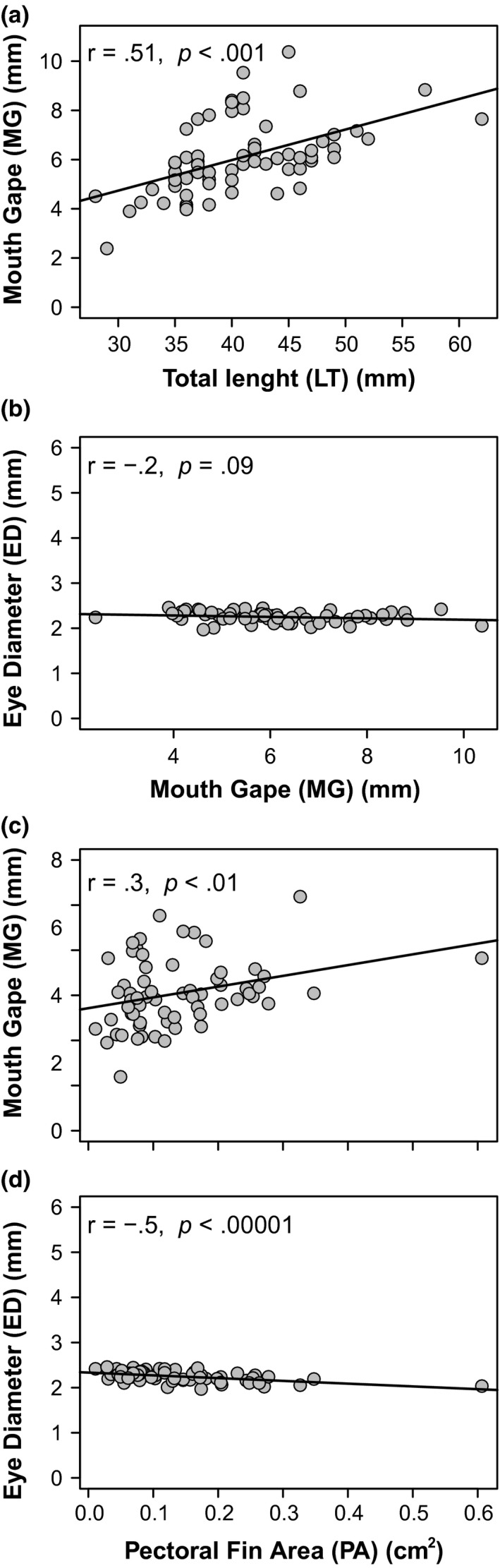
Relationship between size‐corrected morphological measurements (allometry removed) of (a) mouth gape and total length, (b) eye diameter and mouth gape, (c) mouth gape and pectoral fin area, and d) eye diameter and pectoral fin area

Using size‐corrected morphological characters, significant positive correlations were observed between TL and mouth gape (MG) and between pectoral fin area (PA) and MG (Figure 4). However, no correlations were observed in size‐corrected values between MG and eye diameter (ED) or between PA and ED, in contrast to the results using un‐corrected data (Figure 4). This underscores the importance of removing allometry when comparing pairs of morphological structures (Figure S3).

### 3.3 Dietary analysis

**Figure 5:**
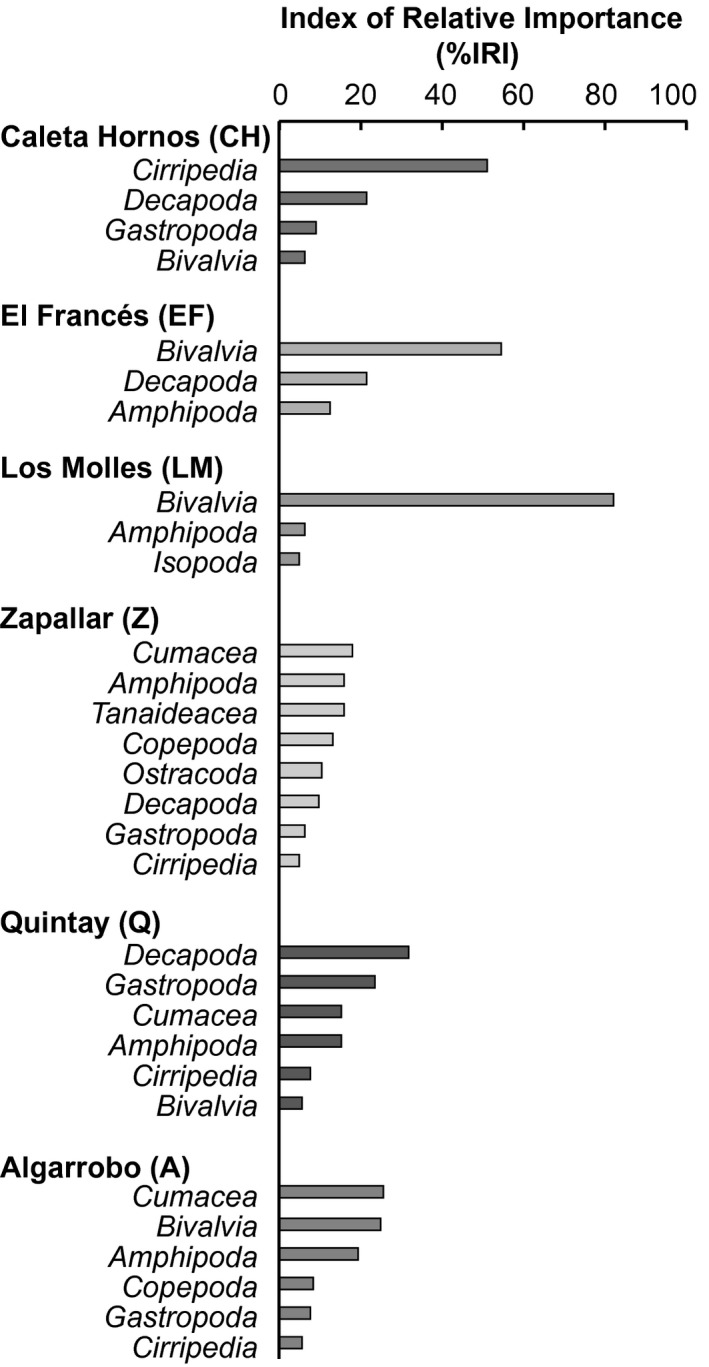
Percent (%) Index of Relative Importance IRI of diet composition among sites

The contribution of each prey item was calculated per sampling site and summarized using the Index of Relative Importance (IRI). The prey categories that contributed with an IRI higher than 5% were considered important in the overall diet of *H. cunninghami* across study sites and included cirripedia, bivalvia, decapoda, and cumacea (Figure 5). However, we observed differences between sites in terms of the relative importance of different prey types and overall diet breadth. For example, while at the three northern sites of CH, EF and LM diet composition was dominated by one important taxonomical group that contributed to more than 50% of the diet (cirripedia, bivalvia, and decapoda, respectively), at the southern sites, fish preyed on a broader range of organisms (including prey from the groups cumacea, amphipoda, cirripedia, bivalvia, copepoda, decapoda, gastropoda, tanaideacea, and ostracoda among others), each of these contributing to less than 30% of the diet composition.

The effect of prey preference on fish morphology differed based on the prey type and morphological structure analyzed. For example, copepods, cumaceans, decapods, hydroids, and ophiruoids were each associated with specific morphological structures (Table 2). However, the direction of change in dietary composition drove differences in the magnitude and direction of variation among morphological structures. Individuals with larger mouth gape (MG) tended to have diets with a greater proportion of decapods (LMM: *t* = 2.98, *p *=* *.003), whereas lower values of MG were associated with copepods (LMM: *t* = −2.7, *p *=* *.006) and cumaceans (LMM: *t* = −2.0, *p *=* *.04). Larger eyes were associated with hydroids (LMM: *t* = 1.99, *p *<* *.05) and cumaceans (LMM: *t* = 2.82, *p *=* *.005), while individuals with smaller eye diameter tended to eat decapods (LMM: *t* = −2.37, *p *<* *.05) and have lower amounts of hydroids in their diets (LMM: *t* = −2.6, *p *=* *.009). Fish with higher fin aspect ratio were associated with ophiuroidea (LMM: *t* = 2.1, *p *=* *.04) but low percent volume of copepods (LMM: *t* = −2.01, *p *<* *.04) and cumaceans (LMM: *t* = −2.4, *p* = .01). Longer pectoral fin length was associated with high percent volume of cumaceans (LMM: *t* = 1.88, *p *=* *.05). Finally, higher values of pectoral fin area were positively associated with decapods (LMM: *t* = 2.45, *p *<* *.05) but negatively with cumaceans (LMM: *t* = −3.4, *p *<* *.001) (Table 2).

**Table 2:** Linear Mixed model effect on size (total length)‐corrected morphological structures and volumetric composition of different prey items. Bold values indicate statistical significance at *p*‐values ≤.05

Morphological Characteristics	Statistics	Prey category															
		Bivalvia	Copepoda	Tanaideacea	Amphipoda	Cirripedia	Cumacea	Gastropoda	Decapoda	Ostracoda	Isopoda	Hydrozoa	Polychaeta	Crustacea	Vermetidae	Ophiuroidea	Mollusca
Mouth Gape (MG)	*t* value	−0.453	−2.702	0.277	−1.028	−1.433	−2.002	1.081	2.968	−0.011	−0.156	0.916	−0.758	−1.507	0.139	0.738	1.483
	Pr(>Chisq)	0.65	**0.006**	0.782	0.304	0.152	**0.04**	0.279	**0.003**	0.991	0.876	0.359	0.448	0.132	0.889	0.46	0.138
	Site variance	0.194	0.214	0.182	0.157	0.190	0.094	0.192	0.113	0.176	0.178	0.143	0.144	0.234	0.177	0.188	0.160
Eye diameter (ED)	*t* value	0.48	0.918	0.2	0.73	1.7	2.82	−1.14	−2.37	0.42	−0.20	1.99	0.95	1.13	0.24	−2.6	0.68
	Pr(>Chisq)	0.628	0.359	0.838	0.463	0.088	**<0.005**	0.255	**<0.05**	0.672	0.843	**0.046**	0.344	0.259	0.812	**0.009**	0.498
	Site variance	0.181	0.000	0.154	0.183	0.111	0.282	0.210	0.170	0.154	0.153	0.272	0.190	0.138	0.149	0.231	0.135
Fin aspect ratio (AR)	*t* value	−1.344	−2.018	−0.192	−0.16	−0.915	−2.339	1.710	0.82	−0.162	0.851	1.44	−0.103	−0.97	−0.737	2.046	−0.702
	Pr(>Chisq)	0.179	**0.043**	0.848	0.872	0.36	**0.019**	0.087	0.412	0.871	0.395	0.15	0.918	0.332	0.461	**0.04**	0.482
	Site variance	0.078	0.218	0.072	0.078	0.066	0.155	0.090	0.057	0.072	0.090	0.051	0.079	0.068	0.075	0.100	0.073
Pectoral fin length (PL)	*t* value	0.933	0.408	0.672	0.529	0.858	1.881	−1.86	−0.844	0.061	−0.156	−1.507	−0.175	0.327	1.165	−1.692	1.062
	Pr(>Chisq)	0.35	0.683	0.502	0.596	0.391	**0.05**	0.062	0.398	0.951	0.129	0.131	0.861	0.743	0.244	0.09	0.288
	Site variance	0.150	0.156	0.115	0.136	0.123	0.112	0.161	0.092	0.133	0.000	0.074	0.134	0.137	0.121	0.178	0.155
Pectoral fin area (PA)	*t* value	−0.937	−1.932	−0.275	−1.046	−1.449	−3.408	1.242	2.455	−0.443	−0.382	−0.187	−0.026	−1.482	−0.294	0.991	−0.458
	Pr(>Chisq)	0.349	0.053	0.783	0.295	0.147	**<0.001**	0.214	**<0.05**	0.658	0.702	0.852	0.979	0.138	0.7691	0.321	0.646
	Site variance	0.106	0.131	0.112	0.147	0.133	0.344	0.095	0.148	0.109	0.108	0.117	0.118	0.095	0.124	0.092	0.111

### 3.4 Multivariate analysis

Diet composition differed significantly among sites (PERMANOVA, Pseudo‐*F*_5,64_ = 5.08, *P*(Perm) = 0.0001). According to SIMPER analysis, dietary items that contributed the most to this difference were bivalvia (20.54%), copepoda (13.23%), tanaideacea (12.2%), amphipoda (11.85%), and cirripedia (11.56%) (Table 3).

**Table 3:** Mean contribution (percentage ± *SD*) of prey categories from SIMPER dissimilarity analysis of prey volume (%V) between sampling sites [Caleta Hornos (CH), El Francés (EF), Los Molles (LM), Zapallar (Z), Quintay (Q), and Algarrobo (A)]. For detailed information about the analysis per contrasting site see Table S3

Prey category	Mean contrib% ± *SD*
Bivalvia	20.54 ± 12.54
Copepoda	13.23 ± 3.74
Tanaideacea	12.20 ± 9.09
Amphipoda	11.85 ± 3.37
Cirripedia	11.56 ± 8.25
Cumacea	9.83 ± 3.31
Gastropoda	8.52 ± 3.25
Decapoda	7.85 ± 3.36
Ostracoda	4.78 ± 1.79
Isopoda	4.58 ± 1.63
Hydrozoa	4.36 ± 1.09
Polychaeta	4.21 ± 0.50
Crustacea	3.15 ± 0.45

## 4 DISCUSSION

We have described how the distribution of microhabitats and the feeding resources they offer influence variation in the diet and morphological structures used for foraging by a small temperate reef fish (*H. cunninghami*). This common triplefin occupies different microhabitats and consumes different prey types throughout its distribution in northern‐central Chile. We observed a close relationship between this variation in habitat/diet selection and key feeding morphological structures. This correlation between variation in foraging behavior and morphological structures suggests that polymorphisms are a mechanism for this species to thrive on the heterogeneous temperate rocky reefs found throughout its range.

*Helcogrammoides cunninghami* feeds mostly upon sessile benthic invertebrates such as bivalvia, which were the most abundant groups overall in gut content analyses, followed by cumacea, amphipoda, cirripedia, copepoda, decapoda, gastropoda, tanaideacea, and ostracoda. Our data are in accordance with other studies of dietary composition and trophic position for this cryptic microcarnivore (Feary et al., 2009). However, one of our most striking findings was that predation was focused on one prey type at half of our sites, while at other sites, prey usage was much more even across several prey categories. A spatial pattern emerged from gut content analysis with preference for either cirripedia or bivalvia at more northern sites, in contrast to a diverse prey content including cumacea, amphipoda, cirripedia, bivalvia, copepoda, decapoda, gastropoda, tanaideacea, and ostracoda at southern sites. Bivalivia and cirripedia were the only two groups that were in both the higher IRI categories for importance in diet and the higher SIMPER categories of important species for differences in multivariate diet between sites, indicating that barnacle and bivalve distributions may be the strongest prey category drivers of diet and foraging morphology for *H. cunninghami*. It is unclear whether this is due to an underlying latitudinal gradient in prey preference or represents a shift in the prey available in understory habitats. According to (Pérez‐Matus et al., 2016), understory benthic species are important in explaining the abundance of *H. cunninghami* in kelp‐dominated habitats, as these microhabitats serve as both potential foraging sites and as a refuge from predators. Thus, the distribution of microhabitats and associated fauna as potential prey may explain the observed differences in the diet of *H. cunninghami*. To that end, the broader range of habitat and food types available in our southern sites would be predicted to result in more generalist strategies and plastic phenotypes than in the relatively homogeneous northern sites.

At all three northern sites (CH, EF, and LM), triplefins showed strong preference for one prey type (cirripedia or bivalvia). Even though crustose coralline algae were the dominant benthic cover type across all sites (a common feature of understory communities in habitats dominated by macroalgae; Melville & Connell, 2001; Connell, 2003), at LM, there was also 16% macroalgal understory (high compared to other sites). This understory, composed of corticated, foliose, and filamentous algal types, provides substantial microhabitat and foraging space for triplefins. A previous study showed that *H. cunninghami* prefers corticated foliose algae among all habitat types available at LM (Pérez‐Matus et al., 2016). Interestingly, bivalvia species constituted more than 80% of triplefin IRI at this location. Bivalves are often associated with understory algae due to facilitated settlement (Ackerman, Sim, Nichols, & Claudi, 1994; Sunila, 1981). Bivalves were also the preferred food type at El Francés, although algal understory was somewhat less abundant (6%). The northernmost site in our study (CH) stood out from all other sites in that there were no algae available as understory microhabitat. Barnacles (cirripedia) were the most important prey item here, but nowhere else. It is probable that bivalves and amphipods are present in very low abundances at CH, as they need to associate with understory algae in order to settle (Ackerman et al., 1994; Sunila, 1981) and feed (Goecker & Kåll, 2003; Poore, 1994). Pérez‐Matus et al. (2016) found no association by *H. cunninghami* with any specific habitat type at CH (called El Arrayan in the 2016 study). We conclude that the low diversity of microhabitats at northern sites resulted in low prey diversity, which led to dominance of the IRI by singularly abundant prey types: bivalvia at LM and EF, cirripedia at CH.

We also observed an interesting relationship between two cranial structures (mouth gape and eye diameter) and pectoral fin area. This relationship is important given that there is a direct link between eye diameter, the retina, and lens in providing the sensitivity and skill to capture prey at low light intensities (Protas, Conrad, Gross, Tabin, & Borowsky, 2007). Our results suggest that *H. cunninghami* uses both cranial (mouth and eyes) and fin structures (pectoral fins) while foraging, which positions them as active foragers, a trait previously described for this genus (Kotrschal & Thomson, 1986). Fin aspect ratio did not differ among sites and was relatively low (Wainwright et al., 2002), indicating a preferred use of the rowing mode of swimming (Fulton, Bellwood, & Wainwright, 2001; Walker & Westneat, 2000), which is best suited to producing drag‐based paddling (Vogel, 1994; Walker & Westneat, 2000). Labrid fishes (wrasses and parrotfish) with lower aspect ratio paddle‐shaped fins tend to swim more slowly (Wainwright et al., 2002) and occupy less energetic zones on the reef (Bellwood & Wainwright, 2001; Fulton et al., 2001). *H. cunninghami* tends to live in low‐flow habitats, usually a deeper portion of the subtidal margins between 10 and 15 m depth where kelp tends to be sparse (Pérez‐Matus et al., 2016). Binning and Roche (2015) found that even intraspecific fin shape polymorphisms vary with exposure to wave action, such that fish with elongated fins can be found at high wave exposure and individuals with rounder fins prefer sheltered reefs. Thus considerable phenotypic divergence can be observed in response to different habitat conditions and prey types available even over very small spatial scales.

Forging these links between dietary and habitat preferences and feeding structures helps us to understand how the different aspects of species biology, ecology, and relationships with the surrounding environment are correlated with morphology (Pérez‐Matus et al., 2007; Sih & Christensen, 2001). Feeding strategies encompass the decisions of where, when, and what to feed on and are ultimately driven by selection for behavioral and/or morphological adaptations to increase the energy consumed per unit of time (MacArthur & Pianka, 1966). In complex temperate reefs, the type of habitat a given species of fish inhabits determines the use of food resources, as evidence suggests that prey availability determines the diet of the associated species (Angel & Ojeda, 2001; Pérez‐Matus et al., 2012). We found support for our hypothesis that there are driving forces specific to each site (such as availability of foraging habitats, available prey, exposure, kelp density, predation see Pérez‐Matus et al., 2016) that drive phenotypic divergence in morphologies used for feeding. Individuals with larger mouth gape and pectoral fin area [a large surface area moves more water and has a larger effect on the fish's momentum than a smaller surface area see (Langerhans, 2009)] were positively correlated with a high percent volumetric composition of benthic decapod prey. On the contrary, a high volumetric composition of pelagic cumaceans in the diet was associated with smaller mouth gape, smaller pectoral fin area, and low values of fin aspect ratio. A low value of fin aspect ratio is associated with species that generate thrust during power stroke (Wainwright et al., 2002), which may be necessary to pursue small pelagic mobile prey such as cumaceans. High percent volume of cumaceans and hydroids in the stomach contents of this triplefin was associated with higher values of eye diameter. Hydroids are often found attached to kelp holdfasts and rocky substrate covered with other filamentous algae (APM personal observation), therefore differentiating this prey from a heterogeneous background requires good vision; it is worth noting that we did not detect any algal components in the stomach contents of this species. Lastly, capturing larger ophiuroids (larger components in the diet of this triplefin) required higher fin aspect ratio thus reducing drag relative to lift, lending greater efficiency to capturing this type of benthic prey. Thus, we conclude that in these highly diverse environments with multiple feeding resources and opportunities for *H. cunninghami* to forage throughout its range, variation in feeding structures is correlated with variation in diet at local scales (Ferry‐Graham et al., 2001; Horn & Ferry‐Graham, 2006; Wainwright et al., 2002).

We found substantial variation in feeding structures in response to differential habitat and prey availability throughout the range of *H. cunninghami*. When the prey types available are consistent, such as the singular important prey items in our northern sites, there may be pressure for local adaptation and the variation we observed could have a genetic basis. In contrast, populations of *H. cunninghami* in the southern sites may be under selective pressure for morphological traits that are best suited for consuming a variety of mobile and sessile prey items at the same time, as compared to only sessile prey at the northern sites. This could be expected to yield a capacity for phenotypically plastic development of morphological structures and behaviors best suited to the prey located in the particular microhabitat selected by a triplefin recruit. Dietary and morphological plasticity may considerably help predators to forage in multiple microhabitats such as those found on heterogeneous rocky temperate reefs (Fitzpatrick, Gerberich, Kronenberger, Angeloni, & Funk, 2015). Future studies could test this in our system by cross‐transplanting individuals between sites or rearing larval fish in experimental treatments of different habitat and food types and observing phenotypic divergence, in addition to analysis of genetic connectivity among these different populations of *Helcogrammoides cunningami*.

## CONFLICT OF INTEREST

None declared.

## AUTHOR CONTRIBUTIONS

APM conceptualized the study and MPG conducted laboratory analyses. APM, NSW, and RWL analyzed the data. NSW, MPG, RML, and APM wrote the paper.
