# Supplementary material for: Diet reveals links between morphology and foraging in a cryptic temperate reef fish
Source: Ecol Evol. 2017 Nov 15;7(24):11124–34. doi: 10.1002/ece3.3604 (PMC5743691; doi:10.1002/ece3.3604)
Supplement: Supplementary file 1 [file ECE3-7-11124-s001.docx]

**Supplements**

**Figure S1.** Cumulative curves of prey versus number of digestive tracts for each site from north to south: Caleta Hornos, El Francés, Los Molles, Zapallar, Quintay and Algarrobo.

**Figure S2.** Morphological measurements of **a)** Total Length (TL) (cm ± s.e.), **b)** Weight (W) (gr ± s.e.), **c)** Fin Aspect Ratio (AR), **d)** Pectoral Fin Length (PL) (mm ± s.e.), **e)** Pectoral Fin Area (PA) (cm^2^ ± s.e.), **f)** Mouth Gape (MG) (mm ± s.e.) and **g)** Eye Diameter (ED) (mm ± s.e.), calculated from triplefin individuals at the different sampling sites: Caleta Hornos (CH), El Francés (EF), Los Molles (LM), Zapallar (Z), Quintay (Q) and Algarrobo (A)..

**Figure S3.** Relationship between non size-corrected morphological measurements (with allometry): **a)** mouth gape and total length, **b)** eye diameter and mouth gape, **c)** mouth gape and pectoral fin area, and **d)** eye diameter and pectoral fin area.

**Table S1.** Results of one-way ANOVA analyses of the effect of sampling site on microhabitat cover. The effect on: **(a)** Sessile animal understory, **(b)** Algal understory, **(c)** Sand and **(d)** Crustose algae cover was tested. Significant effects (*p* < 0.05) are highlighted in bold.

| **ONE-WAY ANOVA** | **df** | **SS** | **MS** | **F** | ***p*** |
| --- | --- | --- | --- | --- | --- |
| ***(a) Sessile animal understory*** |  |  |  |  |  |
| Site | 5 | 24.97 | 4.99 | 2.09 | 0.19 |
| Residuals | 6 | 14.33 | 2.38 |  |  |
| Total | 11 | 35.3 |  |  |  |
| ***(b) Algal understory*** |  |  |  |  |  |
| Site | 5 | 20.13 | 4.027 | 6.22 | **0.02** |
| Residuals | 6 | 3.88 | 0.64 |  |  |
| Total | 11 | 24.0 |  |  |  |
| ***(c) Sand*** |  |  |  |  |  |
| Site | 5 | 5.35 | 1.07 | 1 | 0.48 |
| Residuals | 6 | 6.42 | 1.07 |  |  |
| Total | 11 | 11.77 |  |  |  |
| ***(d) Crustose algae*** |  |  |  |  |  |
| Site | 5 | 1.58 | 0.31 | 0.89 | 0.54 |
| Residuals | 6 | 2.12 | 0.35 |  |  |
| Total | 11 | 3.7 |  |  |  |

**Table S2.** Results of one-way ANOVA analyses of the effect of sampling site on morphological characters among triplefins. The effects on: **(a)** Total Length (TL), **(b)** Weight (W), **(c)** Fin Aspect Ratio (AR), **(d)** Pectoral Fin Length (PL), **(e)** Pectoral Fin Area (PA), **(f)** Mouth Gape (MG) and **(g)** Eye Diameter (ED) were tested. Significant effects (*p* < 0.05) are highlighted in bold.

| **ONE-WAY ANOVA** | **df** | **SS** | **MS** | **F** | ***p*** |
| --- | --- | --- | --- | --- | --- |
| ***(a) Total Length (LT)*** |  |  |  |  |  |
| Site | 5 | 0.543 | 0.109 | 0.258 | 0.934 |
| Residuals | 59 | 24.814 | 0.421 |  |  |
| Total | 64 | 25.357 |  |  |  |
| ***(b) Weight (W)*** |  |  |  |  |  |
| Site | 5 | 0.148 | 0.030 | 2.99 | 0.911 |
| Residuals | 59 | 5.846 | 0.099 |  |  |
| Total | 64 | 5.994 |  |  |  |
| ***(c) Fin Aspect Ratio (AR)*** |  |  |  |  |  |
| Site | 5 | 2.403 | 0.480 | 1.651 | 0.161 |
| Residuals | 59 | 17.174 | 0.291 |  |  |
| Total | 64 | 19.577 |  |  |  |
| ***(d) Pectoral Fin Length (PL)*** |  |  |  |  |  |
| Site | 5 | 0.241 | 0.048 | 2.398 | **0.0478** |
| Residuals | 59 | 1.188 | 0.020 |  |  |
| Total | 64 | 1.429 |  |  |  |
| ***(e) Pectoral Fin Area (PA)*** |  |  |  |  |  |
| Site | 5 | 0.099 | 0.019 | 2.265 | 0.059 |
| Residuals | 59 | 0.518 | 0.0088 |  |  |
| Total | 64 | 0.617 |  |  |  |
| ***(f) Mouth Gape (MG)*** |  |  |  |  |  |
| Site | 5 | 28.18 | 5.636 | 2.855 | **0.0225** |
| Residuals | 59 | 116.49 | 1.974 |  |  |
| Total | 64 | 144.67 |  |  |  |
| ***(g) Eye Diameter (ED)*** |  |  |  |  |  |
| Site | 5 | 0.151 | 0.0301 | 2.598 | **0.0344** |
| Residuals | 59 | 0.684 | 0.0116 |  |  |
| Total | 64 | 0.835 |  |  |  |

**Table S3.** Results of SIMPER analysis of dissimilarity of prey volume (%V) of each prey category between sampling sites (Caleta Hornos (CH), El Francés (EF), Los Molles (LM), Zapallar (Z), Quintay (Q) and Algarrobo (A)). Cut-off for cumulative contributions: 90.00%. Av. Abund. a and Av. Abund. b= average abundances per contrasting sites (a-b), Av. Diss. = average dissimilarity, Diss/SD = quotient of dissimilarity and standard deviation, Contrib% = percentage contribution of each prey category to variation between the sites, Cum.% = ordered cumulative contribution in percentage.

| **SIMPER between sites** |  |  |  |  |  |  |  |  |
| --- | --- | --- | --- | --- | --- | --- | --- | --- |
| **Contrasting sites** | **Average dissimilarity** | **Prey category** | **Av. Abund. a** | **Av. Abund. b** | **Av. Diss.** | **Diss/SD** | **Contrib%** | **Cum.%** |
| Caleta Hornos - El Francés | 83.61 | Bivalvia | 0.39 | 2.62 | 25.90 | 1.55 | 30.97 | 30.97 |
|  |  | Cirripedia | 1.87 | 0.00 | 20.26 | 1.49 | 24.23 | 55.20 |
|  |  | Amphipoda | 0.25 | 0.97 | 9.25 | 0.94 | 11.06 | 66.26 |
|  |  | Decapoda | 0.53 | 0.41 | 6.66 | 0.92 | 7.96 | 74.22 |
|  |  | Gastropoda | 0.54 | 0.23 | 5.92 | 0.65 | 7.08 | 81.31 |
|  |  | Copepoda | 0.05 | 0.55 | 5.19 | 0.91 | 6.20 | 87.51 |
|  |  | Isopoda | 0.23 | 0.14 | 2.78 | 1.08 | 3.33 | 90.84 |
| El Francés - Zapallar | 81.72 | Bivalvia | 2.62 | 0.22 | 18.39 | 1.54 | 22.51 | 22.51 |
|  |  | Tanaideacea | 0.00 | 2.60 | 17.26 | 1.22 | 21.12 | 43.63 |
|  |  | Copepoda | 0.55 | 1.81 | 11.29 | 1.26 | 13.81 | 57.44 |
|  |  | Amphipoda | 0.97 | 1.34 | 10.14 | 1.20 | 12.41 | 69.85 |
|  |  | Hydrozoa | 0.22 | 0.55 | 4.42 | 0.77 | 5.40 | 75.26 |
|  |  | Cumacea | 0.00 | 0.62 | 4.34 | 1.55 | 5.31 | 80.56 |
|  |  | Decapoda | 0.41 | 0.30 | 3.61 | 0.84 | 4.42 | 84.98 |
|  |  | Cirripedia | 0.00 | 0.33 | 3.09 | 0.45 | 3.79 | 88.76 |
|  |  | Ostracoda | 0.06 | 0.33 | 2.34 | 0.89 | 2.87 | 91.63 |
| Caleta Hornos - Zapallar | 86.21 | Tanaideacea | 0.03 | 2.60 | 18.44 | 1.22 | 21.39 | 21.39 |
|  |  | Cirripedia | 1.87 | 0.33 | 13.42 | 1.55 | 15.56 | 36.95 |
|  |  | Copepoda | 0.05 | 1.81 | 13.31 | 1.25 | 15.43 | 52.38 |
|  |  | Amphipoda | 0.25 | 1.34 | 10.53 | 1.06 | 12.21 | 64.59 |
|  |  | Cumacea | 0.00 | 0.62 | 4.68 | 1.56 | 5.43 | 70.02 |
|  |  | Gastropoda | 0.54 | 0.28 | 4.64 | 0.69 | 5.38 | 75.40 |
|  |  | Decapoda | 0.53 | 0.30 | 4.52 | 0.89 | 5.24 | 80.65 |
|  |  | Hydrozoa | 0.05 | 0.55 | 4.25 | 0.68 | 4.93 | 85.58 |
|  |  | Bivalvia | 0.39 | 0.22 | 3.82 | 0.72 | 4.43 | 90.01 |
| El Francés - Los Molles | 52.66 | Bivalvia | 2.62 | 3.16 | 17.62 | 1.13 | 33.46 | 33.46 |
|  |  | Amphipoda | 0.97 | 0.18 | 8.83 | 0.91 | 16.77 | 50.23 |
|  |  | Copepoda | 0.55 | 0.07 | 5.22 | 0.92 | 9.92 | 60.14 |
|  |  | Decapoda | 0.41 | 0.07 | 4.52 | 0.72 | 8.58 | 68.73 |
|  |  | Ostracoda | 0.06 | 0.35 | 4.29 | 0.47 | 8.15 | 76.88 |
|  |  | Isopoda | 0.14 | 0.23 | 3.48 | 0.46 | 6.60 | 83.48 |
|  |  | Gastropoda | 0.23 | 0.06 | 2.31 | 0.96 | 4.39 | 87.87 |
|  |  | Hydrozoa | 0.22 | 0.00 | 1.93 | 0.48 | 3.67 | 91.54 |
| Caleta Hornos - Los Molles | 87.74 | Bivalvia | 0.39 | 3.16 | 33.17 | 1.93 | 37.80 | 37.80 |
|  |  | Cirripedia | 1.87 | 0.02 | 22.23 | 1.50 | 25.34 | 63.14 |
|  |  | Decapoda | 0.53 | 0.07 | 6.01 | 0.78 | 6.85 | 69.99 |
|  |  | Gastropoda | 0.54 | 0.06 | 5.81 | 0.55 | 6.62 | 76.61 |
|  |  | Ostracoda | 0.07 | 0.35 | 4.97 | 0.50 | 5.67 | 82.28 |
|  |  | Isopoda | 0.23 | 0.23 | 4.57 | 0.58 | 5.21 | 87.49 |
|  |  | Amphipoda | 0.25 | 0.18 | 3.04 | 0.87 | 3.47 | 90.96 |
| Zapallar - Los Molles | 90.90 | Bivalvia | 0.22 | 3.16 | 23.11 | 1.82 | 25.42 | 25.42 |
|  |  | Tanaideacea | 2.60 | 0.17 | 17.58 | 1.20 | 19.34 | 44.77 |
|  |  | Copepoda | 1.81 | 0.07 | 13.11 | 1.24 | 14.42 | 59.19 |
|  |  | Amphipoda | 1.34 | 0.18 | 10.49 | 1.07 | 11.55 | 70.73 |
|  |  | Cumacea | 0.62 | 0.00 | 4.63 | 1.57 | 5.09 | 75.83 |
|  |  | Ostracoda | 0.33 | 0.35 | 4.47 | 0.74 | 4.92 | 80.75 |
|  |  | Hydrozoa | 0.55 | 0.00 | 4.04 | 0.63 | 4.45 | 85.19 |
|  |  | Cirripedia | 0.33 | 0.02 | 3.42 | 0.47 | 3.77 | 88.96 |
|  |  | Decapoda | 0.30 | 0.07 | 2.46 | 0.72 | 2.70 | 91.66 |
| El Francés - Quintay | 82.22 | Bivalvia | 2.62 | 0.33 | 24.23 | 1.54 | 29.47 | 29.47 |
|  |  | Amphipoda | 0.97 | 0.71 | 9.54 | 1.18 | 11.60 | 41.07 |
|  |  | Decapoda | 0.41 | 0.93 | 9.36 | 0.88 | 11.39 | 52.46 |
|  |  | Gastropoda | 0.23 | 1.03 | 8.98 | 0.99 | 10.92 | 63.39 |
|  |  | Cumacea | 0.00 | 0.90 | 8.16 | 0.94 | 9.93 | 73.32 |
|  |  | Cirripedia | 0.00 | 0.59 | 6.07 | 0.61 | 7.38 | 80.69 |
|  |  | Copepoda | 0.55 | 0.05 | 4.92 | 0.90 | 5.98 | 86.67 |
|  |  | Crustacea | 0.11 | 0.18 | 2.34 | 0.46 | 2.85 | 89.52 |
|  |  | Hydrozoa | 0.22 | 0.00 | 1.82 | 0.48 | 2.21 | 91.73 |
| Caleta Hornos - Quintay | 76.55 | Cirripedia | 1.87 | 0.59 | 17.93 | 1.42 | 23.43 | 23.43 |
|  |  | Gastropoda | 0.54 | 1.03 | 11.75 | 1.05 | 15.35 | 38.78 |
|  |  | Decapoda | 0.53 | 0.93 | 10.91 | 0.94 | 14.25 | 53.03 |
|  |  | Cumacea | 0.00 | 0.90 | 9.03 | 0.95 | 11.80 | 64.83 |
|  |  | Amphipoda | 0.25 | 0.71 | 6.80 | 1.02 | 8.88 | 73.71 |
|  |  | Bivalvia | 0.39 | 0.33 | 5.81 | 0.75 | 7.59 | 81.30 |
|  |  | Crustacea | 0.14 | 0.18 | 2.92 | 0.52 | 3.82 | 85.11 |
|  |  | Isopoda | 0.23 | 0.00 | 2.43 | 0.99 | 3.18 | 88.29 |
|  |  | Tanaideacea | 0.03 | 0.19 | 2.03 | 0.45 | 2.65 | 90.94 |
| Zapallar - Quintay | 79.71 | Tanaideacea | 2.60 | 0.19 | 16.70 | 1.16 | 20.95 | 20.95 |
|  |  | Copepoda | 1.81 | 0.05 | 12.51 | 1.23 | 15.69 | 36.65 |
|  |  | Amphipoda | 1.34 | 0.71 | 9.68 | 1.23 | 12.14 | 48.79 |
|  |  | Decapoda | 0.30 | 0.93 | 6.90 | 0.84 | 8.66 | 57.45 |
|  |  | Gastropoda | 0.28 | 1.03 | 6.64 | 0.99 | 8.34 | 65.78 |
|  |  | Cumacea | 0.62 | 0.90 | 6.05 | 1.21 | 7.59 | 73.37 |
|  |  | Cirripedia | 0.33 | 0.59 | 5.89 | 0.76 | 7.38 | 80.76 |
|  |  | Hydrozoa | 0.55 | 0.00 | 3.85 | 0.63 | 4.82 | 85.58 |
|  |  | Bivalvia | 0.22 | 0.33 | 3.19 | 0.70 | 4.00 | 89.58 |
|  |  | Crustacea | 0.18 | 0.18 | 2.39 | 0.49 | 3.00 | 92.58 |
| Los Molles - Quintay | 89.56 | Bivalvia | 3.16 | 0.33 | 30.83 | 1.88 | 34.42 | 34.42 |
|  |  | Gastropoda | 0.06 | 1.03 | 10.27 | 0.99 | 11.47 | 45.89 |
|  |  | Decapoda | 0.07 | 0.93 | 9.93 | 0.79 | 11.09 | 56.98 |
|  |  | Cumacea | 0.00 | 0.90 | 8.90 | 0.95 | 9.93 | 66.92 |
|  |  | Cirripedia | 0.02 | 0.59 | 6.75 | 0.63 | 7.54 | 74.46 |
|  |  | Amphipoda | 0.18 | 0.71 | 6.21 | 0.94 | 6.93 | 81.39 |
|  |  | Ostracoda | 0.35 | 0.00 | 4.08 | 0.42 | 4.56 | 85.95 |
|  |  | Tanaideacea | 0.17 | 0.19 | 3.08 | 0.66 | 3.44 | 89.39 |
|  |  | Crustacea | 0.10 | 0.18 | 2.61 | 0.45 | 2.92 | 92.30 |
| El Francés - Algarrobo | 76.31 | Bivalvia | 2.62 | 0.65 | 19.44 | 1.52 | 25.47 | 25.47 |
|  |  | Copepoda | 0.55 | 1.44 | 11.80 | 1.08 | 15.46 | 40.93 |
|  |  | Amphipoda | 0.97 | 1.30 | 11.69 | 1.12 | 15.32 | 56.25 |
|  |  | Cumacea | 0.00 | 1.19 | 10.18 | 1.01 | 13.34 | 69.59 |
|  |  | Gastropoda | 0.23 | 0.55 | 5.28 | 0.71 | 6.92 | 76.51 |
|  |  | Polychaeta | 0.07 | 0.30 | 3.56 | 0.35 | 4.66 | 81.17 |
|  |  | Decapoda | 0.41 | 0.09 | 3.46 | 0.68 | 4.53 | 85.70 |
|  |  | Cirripedia | 0.00 | 0.38 | 3.35 | 0.65 | 4.39 | 90.09 |
| Caleta Hornos - Algarrobo | 83.50 | Cirripedia | 1.87 | 0.38 | 16.37 | 1.43 | 19.60 | 19.60 |
|  |  | Copepoda | 0.05 | 1.44 | 12.45 | 0.90 | 14.90 | 34.51 |
|  |  | Amphipoda | 0.25 | 1.30 | 11.26 | 0.96 | 13.49 | 47.99 |
|  |  | Cumacea | 0.00 | 1.19 | 11.18 | 1.01 | 13.39 | 61.39 |
|  |  | Gastropoda | 0.54 | 0.55 | 7.93 | 0.77 | 9.50 | 70.89 |
|  |  | Bivalvia | 0.39 | 0.65 | 6.69 | 1.09 | 8.02 | 78.91 |
|  |  | Decapoda | 0.53 | 0.09 | 4.75 | 0.77 | 5.68 | 84.59 |
|  |  | Polychaeta | 0.00 | 0.30 | 3.61 | 0.31 | 4.32 | 88.91 |
|  |  | Tanaideacea | 0.03 | 0.30 | 2.83 | 0.68 | 3.39 | 92.29 |
| Zapallar - Algarrobo | 70.84 | Tanaideacea | 2.60 | 0.30 | 15.01 | 1.13 | 21.19 | 21.19 |
|  |  | Copepoda | 1.81 | 1.44 | 12.05 | 1.32 | 17.02 | 38.21 |
|  |  | Amphipoda | 1.34 | 1.30 | 10.38 | 1.23 | 14.66 | 52.87 |
|  |  | Cumacea | 0.62 | 1.19 | 6.77 | 1.07 | 9.56 | 62.42 |
|  |  | Bivalvia | 0.22 | 0.65 | 4.52 | 1.13 | 6.38 | 68.81 |
|  |  | Cirripedia | 0.33 | 0.38 | 4.26 | 0.74 | 6.02 | 74.82 |
|  |  | Gastropoda | 0.28 | 0.55 | 4.13 | 0.76 | 5.83 | 80.65 |
|  |  | Hydrozoa | 0.55 | 0.00 | 3.56 | 0.62 | 5.02 | 85.67 |
|  |  | Polychaeta | 0.00 | 0.30 | 2.39 | 0.31 | 3.37 | 89.04 |
|  |  | Ostracoda | 0.33 | 0.00 | 2.17 | 0.82 | 3.06 | 92.10 |
| Los Molles - Algarrobo | 85.56 | Bivalvia | 3.16 | 0.65 | 25.71 | 2.12 | 30.05 | 30.05 |
|  |  | Copepoda | 0.07 | 1.44 | 12.32 | 0.90 | 14.40 | 44.45 |
|  |  | Cumacea | 0.00 | 1.19 | 11.03 | 1.01 | 12.89 | 57.34 |
|  |  | Amphipoda | 0.18 | 1.30 | 10.98 | 0.94 | 12.84 | 70.18 |
|  |  | Gastropoda | 0.06 | 0.55 | 5.38 | 0.62 | 6.29 | 76.47 |
|  |  | Cirripedia | 0.02 | 0.38 | 3.68 | 0.67 | 4.31 | 80.78 |
|  |  | Ostracoda | 0.35 | 0.00 | 3.61 | 0.42 | 4.22 | 84.99 |
|  |  | Polychaeta | 0.01 | 0.30 | 3.59 | 0.32 | 4.19 | 89.19 |
|  |  | Tanaideacea | 0.17 | 0.30 | 3.41 | 0.83 | 3.98 | 93.17 |
| Quintay - Algarrobo | 75.16 | Copepoda | 0.05 | 1.44 | 11.64 | 0.90 | 15.49 | 15.49 |
|  |  | Amphipoda | 0.71 | 1.30 | 10.79 | 1.11 | 14.35 | 29.85 |
|  |  | Cumacea | 0.90 | 1.19 | 10.32 | 1.16 | 13.73 | 43.57 |
|  |  | Gastropoda | 1.03 | 0.55 | 9.53 | 1.08 | 12.68 | 56.25 |
|  |  | Decapoda | 0.93 | 0.09 | 8.06 | 0.78 | 10.73 | 66.98 |
|  |  | Cirripedia | 0.59 | 0.38 | 6.86 | 0.85 | 9.12 | 76.10 |
|  |  | Bivalvia | 0.33 | 0.65 | 6.06 | 1.10 | 8.06 | 84.16 |
|  |  | Tanaideacea | 0.19 | 0.30 | 3.45 | 0.74 | 4.59 | 88.75 |
|  |  | Polychaeta | 0.02 | 0.30 | 3.37 | 0.32 | 4.49 | 93.24 |
